# Pigs with δ-sarcoglycan deficiency exhibit traits of genetic cardiomyopathy

**DOI:** 10.1038/s41374-020-0406-7

**Published:** 2020-02-14

**Authors:** Hitomi Matsunari, Michiyo Honda, Masahito Watanabe, Satsuki Fukushima, Kouta Suzuki, Shigeru Miyagawa, Kazuaki Nakano, Kazuhiro Umeyama, Ayuko Uchikura, Kazutoshi Okamoto, Masaki Nagaya, Teruhiko Toyo-oka, Yoshiki Sawa, Hiroshi Nagashima

**Affiliations:** 1 Meiji University International Institute for Bio-Resource Research, Kawasaki, 214-8571 Japan; 2 Laboratory of Developmental Engineering, Department of Life Sciences, School of Agriculture, Meiji University, Kawasaki, 214-8571 Japan; 3 Department of Cardiovascular Surgery, Osaka University Graduate School of Medicine, Suita, 565-0871 Japan; 4 Department of Cardioangiology, Kitasato University, Sagamihara, 252-0375 Japan

**Keywords:** Genetic engineering, Genetic engineering

## Abstract

Genetic cardiomyopathy is a group of intractable cardiovascular disorders involving heterogeneous genetic contribution. This heterogeneity has hindered the development of life-saving therapies for this serious disease. Genetic mutations in dystrophin and its associated glycoproteins cause cardiomuscular dysfunction. Large animal models incorporating these genetic defects are crucial for developing effective medical treatments, such as tissue regeneration and gene therapy. In the present study, we knocked out the δ-sarcoglycan (δ-SG) gene (*SGCD*) in domestic pig by using a combination of efficient de novo gene editing and somatic cell nuclear transfer. Loss of δ-SG expression in the *SGCD* knockout pigs caused a concomitant reduction in the levels of α-, β-, and γ-SG in the cardiac and skeletal sarcolemma, resulting in systolic dysfunction, myocardial tissue degeneration, and sudden death. These animals exhibited symptoms resembling human genetic cardiomyopathy and are thus promising for use in preclinical studies of next-generation therapies.

## Abstract (web summary)

Gene δ-sarcoglycan was knocked out in pigs via gene editing and somatic cell cloning. Loss of expression led to α-, β-, and γ-sarcoglycan depletion in the cardiac and skeletal sarcolemma. Pigs exhibiting systolic dysfunction, myocardial tissue degeneration, and sudden death are promising for studying next-generation therapies for human genetic cardiomyopathy.

## Introduction

Idiopathic cardiomyopathy is a serious cardiac disorder manifesting life-threatening regressive lesions of the heart muscle [1]. The burden of this disease globally affects populations across all ages, sexes, and ethnic groups [2]. Genetic cardiomyopathy is a particular group of idiopathic cardiomyopathy, including hypertrophic cardiomyopathy, dilated cardiomyopathy (DCM), arrhythmogenic right ventricular cardiomyopathy, left ventricular noncompaction, and restrictive cardiomyopathy [1, 3]. The involvement of heterogeneous genetic contribution [4] has hindered the development of pharmaceutical agents and therapies specific to genetic cardiomyopathy [5]. Although next-generation therapies, including gene therapy and cell-based therapy, hold promise for curing this intractable disease [6–11], availability of animal models for genetic cardiomyopathy is vital for developing these novel therapies [12–14]. The importance of disease model pigs has been well recognized in recent years, because of the physiological and anatomical similarities between pigs and humans [15–17]. Therefore, we sought to generate swine that exhibit genetic cardiomyopathy.

Monogenic disorder is a promising target for developing model pigs for cardiomyopathy by using currently available technologies [18–21]. Here, we focused on δ-sarcoglycan (δ-SG) gene that causes genetic cardiomyopathy in hamsters [22, 23] and mice [24, 25]. In hamsters of the TO-2 strain carrying the spontaneous biallelic mutation of the δ-SG gene (*SGCD*), DCM is highly prevalent [22, 23]. Genetically engineered Sgcd^−/−^ mice also display many of the important features of DCM, such as left ventricular dilation, thus being a useful preclinical research tool [24, 25]. In contrast to the wealth of mutant laboratory rodents, the number of pig strains with targeted mutations is still inadequate [26] mainly due to technical limitations, particularly the lack of a gene-knockout (KO) strategy involving the use of embryonic stem cells. However, recent advances in genome editing technologies have enabled the generation of KO pigs with increased efficiency [27–29]. In the present study, we knocked out *SGCD* in porcine cells by using transcription activator-like effector nucleases (TALENs) and produced genetically modified cloned animals by using somatic cell nuclear transfer (SCNT). We found that homozygous KO of the *SGCD* in pigs induces a severe phenotype of genetic cardiomyopathy.

## Materials and methods

### Study design

First, we investigated whether biallelic frameshift mutation of *SGCD* causes genetic cardiomyopathy in pigs. Pigs carrying the *SGCD*^*−/−*^ trait were generated via somatic cell cloning, and their phenotypes regarding pathological, physiological, and biochemical features were examined. After confirming the outcome of the *SGCD*^−^^*/−*^ mutation, we generated founder *SGCD*^−/+^ cloned animals and then produced their F2 *SGCD*^−^^/−^ progeny, thereby investigating the reproducibility of the phenotype between cloned founder animals and progeny generated through the sexual reproduction process.

### Animal care and chemicals

The Institutional Animal Care and Use Committee of Meiji University approved all animal experiments (IACUC12-0008, IACUC14-0011). All experiments were performed in accordance with the approved guidelines of the School of Agriculture, Meiji University.

### Direct sequencing of pig *SGCD*

At the onset of the present study the exon−intron structure of porcine *SGCD* had not been reported. Therefore, we first obtained the sequence and structure of the gene, and later verified them on the National Center for Biotechnology Information (NCBI) database (Gene ID 100240724).

Genomic DNA was extracted from the kidney of a female pig (Large White/Landrace × Duroc) by using proteinase K and then purified using the phenol-chloroform method. Genome walking was performed using Genome Walker Universal Kit (Takara Bio, Japan). Briefly, genomic DNA fragments digested with *Dra*I, *Eco*RV, *Pvu*II, and *Stu*I were separately ligated to the Genome Walker Adaptor (provided in the kit) to create genomic DNA libraries. Each library was used as template in the initial PCR amplification with the adaptor-primer AP1 (provided in the kit) and the corresponding primers (SGCD-01−SGCD-08; Table S1). The PCR products were then diluted 1:50 and used in the nested PCR amplification by using the adaptor-primer AP2 (provided in the kit) and the corresponding primers (SGCD-9−SGCD-16; Table S1). The nested PCR products were cloned into the sequencing vector pCR4-Blunt-TOPO (Zero Blunt TOPO PCR Cloning Kit, Life Technologies, USA) and sequenced using the BigDye Terminator Cycle Sequencing Kit (Life Technologies) on the ABI PRISM 3130xl Genetic Analyzer (Life Technologies). The sequences were examined using GENETYX software (Genetyx, Japan) to determine the exon−intron structure of pig *SGCD*.

### Design of TALENs and mRNA preparation

**Fig. 1:**
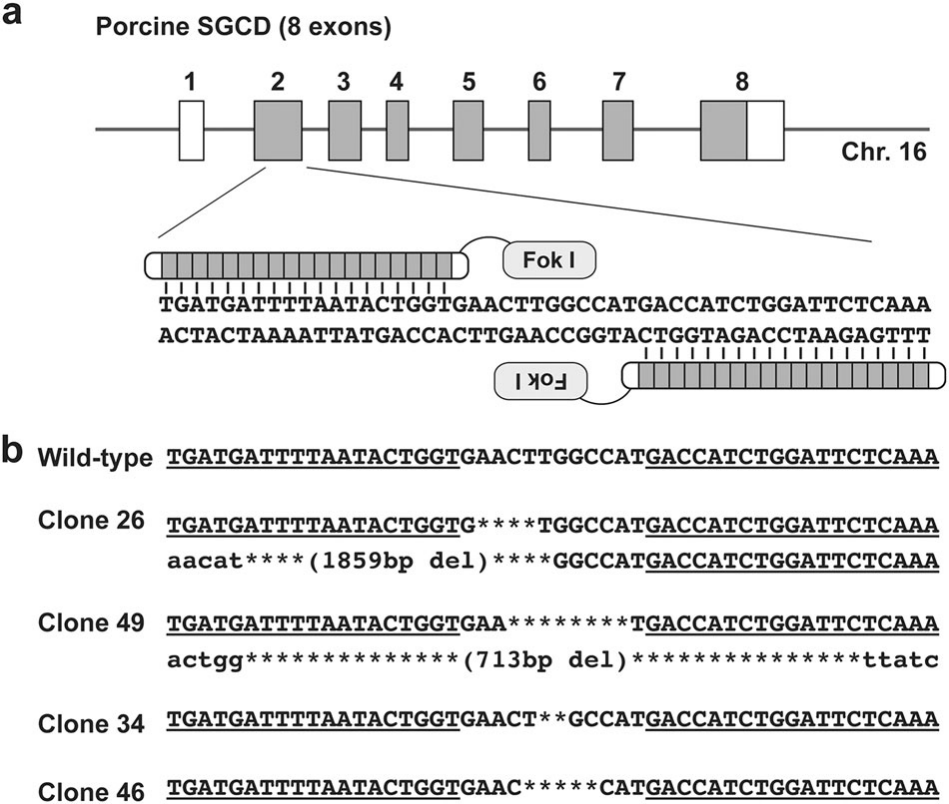
Design of TALENs targeting *SGCD* in PFF cells. **a** Schematic representation of TALENs binding to exon 2 of pig *SGCD*. The coding and untranslated regions of *SGCD* are indicated by the gray and white boxes, respectively. **b** TALEN-induced *SGCD* mutations in cell clones #26, #49, #34, and #46. The deletions are indicated by asterisks. The TALEN-binding sites are underlined.

Custom TALEN plasmids for editing pig *SGCD* were designed and validated by Toolgen Inc. (South Korea) to target the sequence of exon 2 in pig *SGCD* (Fig. 1). The TALEN-encoding mRNAs were produced by linearizing each TALEN plasmid with *Pvu*II followed by purification using phenol-chloroform to generate DNA templates for in vitro transcription. TALEN mRNAs were capped by in vitro transcription by using the MessageMAX T7 ARCA-Capped Message Transcription Kit (Cambio, UK). A Poly(A) tail was then added to each mRNA by using the Poly(A) Polymerase Tailing Kit (Cambio), and the poly(A)-tailed TALEN mRNAs were purified using the spin columns within the MEGAclear Kit (Life Technologies) and resuspended in RNase-free water to 400 ng/µL.

### Establishment of *SGCD*-KO cells

A primary culture of porcine fetal fibroblast (PFF) cells isolated from a male fetus was used to establish the *SGCD*-KO cells. Cells were cultured in MEMα supplemented with 15% fetal bovine serum and 1% antibiotic−antimycotic solution in type-I collagen-coated dishes or plates under a humidified atmosphere containing 5% CO_2_ at 37 °C. For electroporation, the PFF cells were cultured to 70–90% confluence, washed twice with Dulbecco’s phosphate-buffered saline (D-PBS (−)), and detached with 0.05% trypsin-EDTA. These cells (5 × 10^5^) were suspended in 50 μL R buffer (included in the Neon Transfection System, Life Technologies) containing 1 μg of TALEN mRNA. The cells were then electroporated with a single direct current pulse (1100 V, 30 ms; program #6), followed by culture at 32 °C for 3 days without antibiotics for the first 24 h and with antibiotics thereafter [20]. Subsequently, the cells were cultured at 37 °C until they reached 90% confluency, and a limiting dilution was performed to obtain single-cell-derived clones in five 96-well plates. After 14 days, colonies at high confluence (>70%) were selected and divided for subculturing and mutation analysis.

### Determination of TALEN-induced mutations in PFF cells and cloned fetuses

To detect mutations, the target region of *SGCD*-TALENs was amplified by direct PCR of the cell clones by using MightyAmp DNA polymerase Ver.2 (Takara Bio) and corresponding primers (5′-ATGCTCAGAACTTCGTCTCC and 5′-ACTGAGATCTGAAAGAAGTC). Nested PCR was then performed using PrimeSTAR HS DNA polymerase (Takara Bio) and corresponding primers (5′-AAGATGGCTTCCTGCAGGTC and 5′-ACATGTAATGTGAATAGCAGTG). The sequence of the amplicons, including the TALEN target region, was determined using the sequencing primer 5′-TCCTGCAAGTGTCCAGAGGAG and the BigDye Terminator Cycle Sequencing Kit on the ABI PRISM 3130xl Genetic Analyzer.

Large deletions in cell clones #26 and #49 were analyzed by performing nested PCR as described above using the following primers: first round, 5′-ACAGTCAAGCAAGTGGTGCTG and 5′-ACTGAGATCTGAAAGAAGTC; second round, 5′-TCAGTCTCCCAAGGCAACAG and 5′-ACATGTAATGTGAATAGCAGTG. The PCR fragments were cloned into the pCR4-Blunt-TOPO vector for DNA sequencing. The cloned sequences, including the large deletion in clones #26 and #49, were confirmed by DNA sequencing by using the primers 5′-AGATGACCTATGGAGTGGAAG and 5′-AGATCCTGCTGTATAGCACAG, respectively.

Mutations of the cloned pigs and their offspring were analyzed by extracting genomic DNA from tail biopsies using the DNeasy Blood & Tissue Kit (Qiagen, Germany). Genotyping PCR and DNA sequencing were performed as described above. All new sequence data were deposited in the DNA Data Bank of Japan (DDBJ), European Molecular Biology Laboratory (EMBL), and GenBank (NCBI) databases.

### Generation of *SGCD*-mutant pigs by somatic cell cloning

Somatic cell nuclear transfer was performed as previously described [30], with slight modifications. Briefly, *SGCD*^−/−^ or *SGCD*^−/+^ cells were used as nuclear donors following cell cycle synchronization by serum starvation for 2 days. A single donor cell was electrically fused with each enucleated cytoplast prepared from an in-vitro-matured oocyte. The reconstructed embryos were electrically activated and cultured in porcine zygote medium-5 (PZM-5; Research Institute for Functional Peptides, Japan) for 3 h in the presence of 5 μg/mL cytochalasin and 500 nM scriptaid and with 500 nM scriptaid alone for another 12−15 h. After these treatments, the cloned embryos were cultured until transfer to PZM-5 for 1−2 or 5−6 days under a humidified atmosphere of 5% CO_2_, 5% O_2_, and 90% N_2_ at 38.5 °C. After the morula stage was reached, the embryos were cultured in PZM-5 supplemented with 10% fetal bovine serum. The cloned embryos at days 1−2 (2−8 cell stage) or 5−6 (blastocyst stage) were surgically transferred into the oviducts or uterine horns, respectively, of estrus-synchronized recipients.

### Real-time PCR of the skeletal muscle

Nonquantitative expression of each SG transcript was analyzed using real-time PCR (RT-PCR). Total RNA was isolated from pig skeletal muscle by using the RNeasy® Mini Kit (Qiagen), according to the manufacturer’s specifications, and then reverse-transcribed (1 µg) using the Transcriptor First Strand cDNA Synthesis Kit (Roche Diagnostics, Germany), according to the manufacturer’s instructions. The resulting cDNA was then amplified using the primer sets and amplification conditions shown in Table S2 and the Platinum PCR SuperMix (Invitrogen, USA) containing a thermostable DNA polymerase. Levels of the *ACTB* gene (encoding *β*-actin) were used as internal control.

### Antibodies used for biochemical analysis

For western blot and histological analyses, monoclonal mouse antibodies against α-SG (NCL-a-SARC), β-SG (NCL-b-SARC), and γ-SG (NCL-g-SARC) were purchased from Novocastra (Leica Biosystems Newcastle Ltd., UK) and diluted 1:100. A polyclonal site-directed antibody against δ-SG was prepared using a synthetic peptide from the sequence of the cloned δ-SG cDNA and diluted 1:1000 [23]. A monoclonal mouse antibody against the α-spectrin chain (clone AA6) was purchased from Merck Millipore (Germany) and used at a 1:200 dilution. A monoclonal anti-α-tubulin antibody (clone DM 1A) was purchased from Sigma-Aldrich (USA) and used at a 1:10,000 dilution. For immunofluorescence microscopy, Alexa Fluor® 488- or 594-labeled secondary antibodies were obtained from Molecular Probes® (Life Technologies) and diluted to 1:500 before use.

### Western blot analysis of the skeletal muscle

Skeletal muscle tissues were harvested, minced with scissors, homogenized in a tissue grinder (Ultrasonic Homogenizer Smurt NR-50; Microtec, Japan) at 50% output for 30 s with 10 s of rest in three cycles, and solubilized in lysis buffer (CelLytic MT; Sigma-Aldrich). The samples were then centrifuged at 13,000 × *g* for 10 min at 4 °C to remove insoluble debris. The protein concentrations were determined using Bradford’s method [31], and the soluble proteins were resolved using sodium dodecyl sulfate-polyacrylamide gel electrophoresis and transferred to polyvinylidene fluoride membranes. After the blotted membranes were washed with Tween-20/phosphate-buffered saline (PBS), immune complexes were detected using horseradish peroxidase-conjugated anti-rabbit or anti-mouse IgG (DAKO, Denmark) and an ECL kit (GE Healthcare Bio-Sciences, USA).

### Immunofluorescence analysis

Sections of the skeletal and cardiac muscle tissues (10 µm thick) were prepared using a cryostat at –20 °C, fixed in 4% paraformaldehyde for 15 min, and incubated in a solution of PBS with 3% bovine serum albumin for 1 h at approximately 25 °C to reduce background reactivity. Primary antibodies (see above) were diluted in PBS and incubated overnight at 4 °C. Alexa Fluor® 488- or 594-labeled secondary antibody was diluted 1:500 in PBS at approximately 25 °C for 1 h. The sections were mounted with Fluoromount (Diagnostic BioSystems, USA) and observed using a fluorescence microscope.

### Cardiac analysis of *SGCD*^−/−^ pigs and statistical analyses

Resting *SGCD*^−/−^ cloned pigs at 5 weeks of age (*n* = 3) and normal age-matched herdmate controls (*n* *=* 3) not treated with anesthetics or analgesics were subjected to standard transthoracic echocardiography by using a system equipped with a 12 MHz transducer and SONOS 5500 ultrasound (GE Healthcare, USA).

The *SGCD*^*−/−*^ offspring generated from the founder heterozygous *SGCD*-mutant cloned pigs were also examined at 3, 6, and 9 weeks of age (*n* = 2−5 in each group) with their *SGCD*^−/+^ and *SGCD*^+/+^ littermates by using a diagnostic ultrasound system (ARIETTA 70; Hitachi Ltd., Japan) with a 9-2 MHz sector probe.

The diastolic and systolic dimensions of the left ventriculus (LV) were measured using M-mode echocardiography, and the left ventricular ejection fraction (LVEF) was calculated at the same time. The wall thickness of the interventricular septum (IVSth) and posterior LV wall (PWth) was measured using M-mode echocardiography during the diastolic phase. The statistical comparison of the LV systolic dimension and IVSth/PWth between the *SGCD*-KO cloned pigs and the normal age-matched controls was performed using Student’s *t* test, whereas that of the LVEF was performed using Welch’s *t* test. The heart was excised, transversely sliced, fixed by immersion in paraformaldehyde, and embedded in paraffin. Next, 10-μm-thick sections were stained using hematoxylin and eosin (HE) or Masson’s trichrome and examined using an optical microscope.

## Results

### Generation of homozygous *SGCD*-KO cells

Pig *SGCD* was located on chromosome 16 (accession numbers: NW_003301576 and NC_010458) and included an 870-base pair (bp) coding region (accession number: NM_001144123). By using the porcine *SGCD* sequence for DNA sequence analysis, we determined the exon−intron boundaries (Fig. S1). Because *SGCD* in pig includes eight exons with an open reading frame (ORF) starting from exon 2, we constructed a TALEN expression vector to target this exon (Fig. 1).

While establishing *SGCD*-KO cells by using porcine embryonic fibroblasts, we did not observe cellular morphological abnormalities after the introduction of the TALEN-expressing mRNA and the application of transient cold shock. Of the 177 single-cell-derived colonies obtained by limiting dilution, we isolated 67 cell clones (37.9%) with numerous TALEN-induced mutations (Table S3) and established two homozygous *SGCD*-KO cell lines (clones #26 and #49). The mutations included deletions of 4 and 1859 bp in clone #26 and deletions of 8 and 713 bp in clone #49 (Fig. 1). We also generated 29 cell lines, including clones #34 (2 bp deletion) and #46 (5 bp deletion), with various heterozygous frameshift mutations (Fig. 1).

The 4 bp deletion in clone #26 (p.Asn48Trpfs*3) created a stop codon in exon 2, and the 1859 bp deletion removed a large stretch of exon 2, including the start codon upstream of the TALEN cleavage site. The 8 bp deletion in clone #49 (p.Leu49Aspfs*13) created a stop codon between exons 2 and 3, and the 713 bp deletion completely nullified exon 2. We determined that the mutations in clones #26 and #49 led to biallelic inactivation of *SGCD*, and these cells were used as nuclear donors to generate the *SGCD*^−/−^ cloned pigs. Mutations in clones #34 (p.Leu49Cysfs*15) and #46 (p.Leu49Hisfs*14) displayed monoalellic loss-of-function of the gene. These cells were used to generate founder *SGCD*^−/+^ cloned animals.

### Production of *SGCD*^−/−^ cloned pigs

We obtained five offspring after the transfer of 130 *SGCD*^−/−^ cloned embryos at the blastocyst stage into one estrus-synchronized recipient gilt. Of these newborn offspring, two were killed under general anesthesia for analysis. The remaining three offspring survived past the weaning period and underwent transthoracic echocardiography at 5 weeks after birth. One pig died suddenly 7 weeks after birth during body weight measurement, presumably because of a malignant arrhythmia secondary to stress during handling. We conducted echocardiography in the remaining two offspring at 8 weeks after birth; however, they died immediately after the administration of the tranquilizer. These observations suggest the progression of pathology after 5 weeks of age. We assumed that the sudden death was secondary to malignant ventricular arrhythmia or congestive heart failure, which are common manifestations of pediatric/infantile DCM in a clinical scenario. Several specimens from the cadavers were used for analyses.

**Fig. 2:**
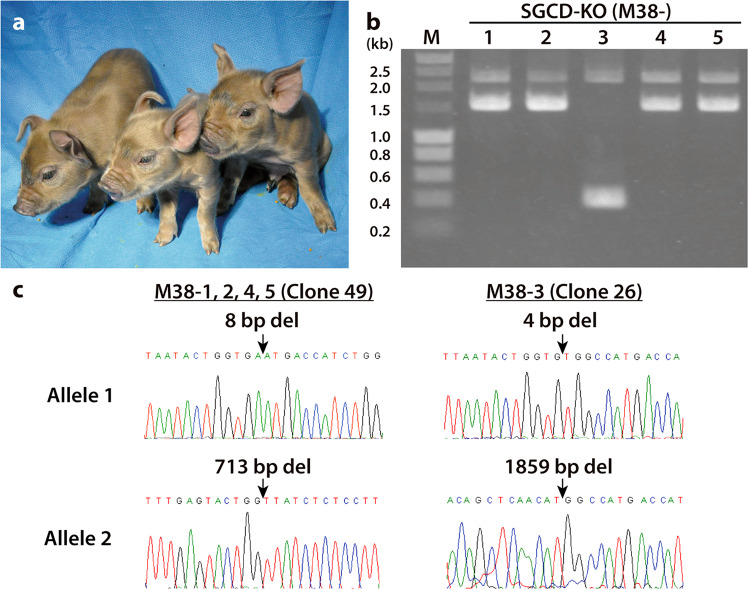
Nucleotide sequence analysis of mutations harbored by *SGCD*-KO pigs. **a** Cloned piglets generated from *SGCD*^−/−^ cells. **b** PCR genotyping of the *SGCD*-KO cloned piglets. Lanes 1, 2, 4, and 5 show 2251 and 1546 bp bands corresponding to the mutated *SGCD* with 8 and 713 bp deletions in nuclear donor cell #49 (Clone #49). Lane 3 shows 2255 and 400 bp bands amplified from nuclear donor cell #26 (Clone #26) harboring 4 and 1859 bp deletions of *SGCD*. M: DNA marker. **c** DNA sequence analysis of *SGCD* in the cloned piglets. The *SGCD* mutations were identical in the cloned piglets and nuclear donor cells (arrows).

Genotyping could be performed using PCRs because of the large deletions within *SGCD* in the cloned pigs. These analyses revealed that, of the five offspring, four (M38-1, 2, 4, and 5) were derived from clone #49 and one (M38-3) was derived from clone #26 (Fig. 2). We confirmed that the offspring and donor cell lines harbored identical *SGCD* mutations (Fig. 2).

### Transcriptional and translational activity of mutant *SGCD*

**Fig. 3:**
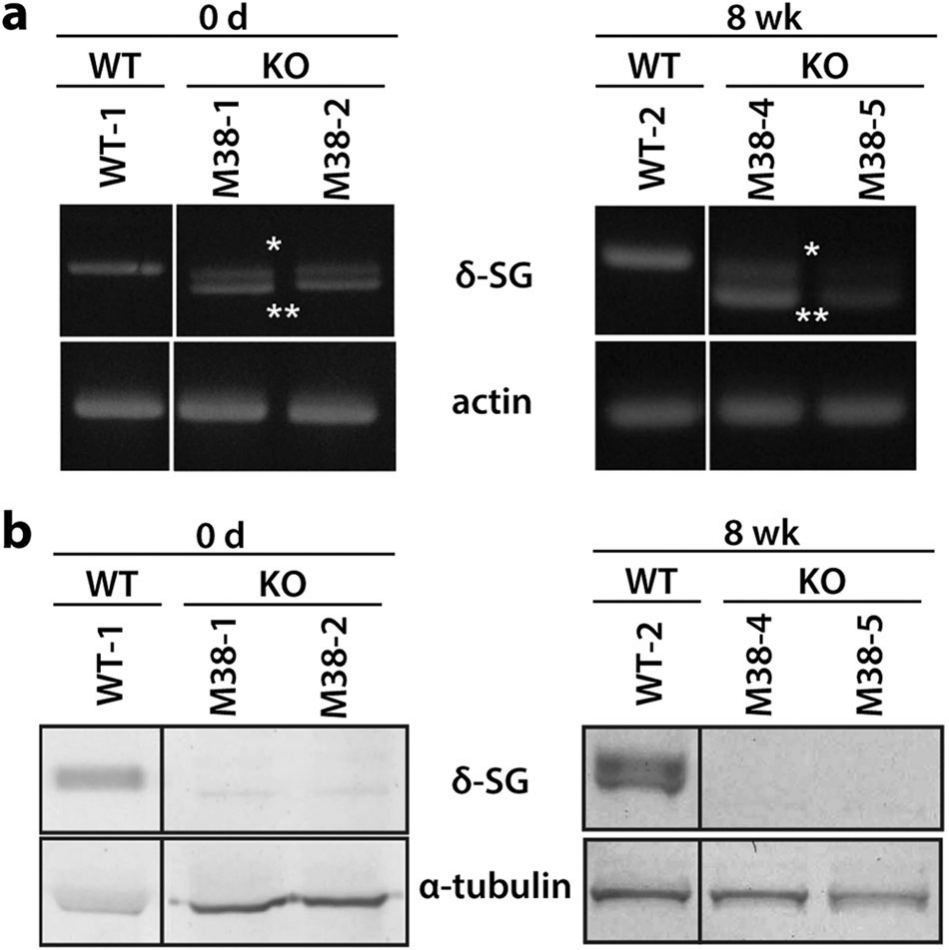
Analysis of *SGCD* expression in the skeletal muscle of *SGCD*^−/−^ pigs. **a** RT-PCR analysis of *SGCD* expression in WT and KO pigs. A 937 bp RT-PCR amplicon was detected in WT tissue, whereas 928 bp (*) and 748 bp (**) amplicons were detected in the KO tissues (M38-1, 2, 4, 5), corresponding to the sizes expected for the mutated alleles. **b** Western blot analysis of the skeletal muscle showed the presence of δ-SG in membrane-enriched skeletal muscle preparations of WT but not of KO pigs, regardless of age. α-Tubulin served as a loading control. Images of cropped gels/blots are shown. Original gels and western blots were run under the same experimental conditions.

Two of the cloned pigs killed on day 0 (M38-1 and M38-2) and two that survived for 8 weeks (M38-4 and M38-5) were derived from clone #49. We analyzed the *SGCD* gene expression and δ-SG protein content in these pig clones. The RT-PCR analysis revealed bands that matched the size of the amplicons generated from the mutant alleles in the skeletal muscle of these four pigs (Fig. 3a). Moreover, the direct sequencing analysis of RT-PCR products revealed exon skipping in the mutant allele that harbored a 713 bp deletion. Western blot analysis of *SGCD*-KO animals on day 0 and at 8 weeks of age revealed δ-SG expression in the skeletal muscle extracts of wild-type (WT) but not *SGCD-*KO pigs (Fig. 3b). These results indicated that the *SGCD-*KO pigs did not express the δ-SG protein, although mutant transcripts were expressed.

### Histological analysis of *SGCD*^−/−^ pig clones

**Fig. 4:**
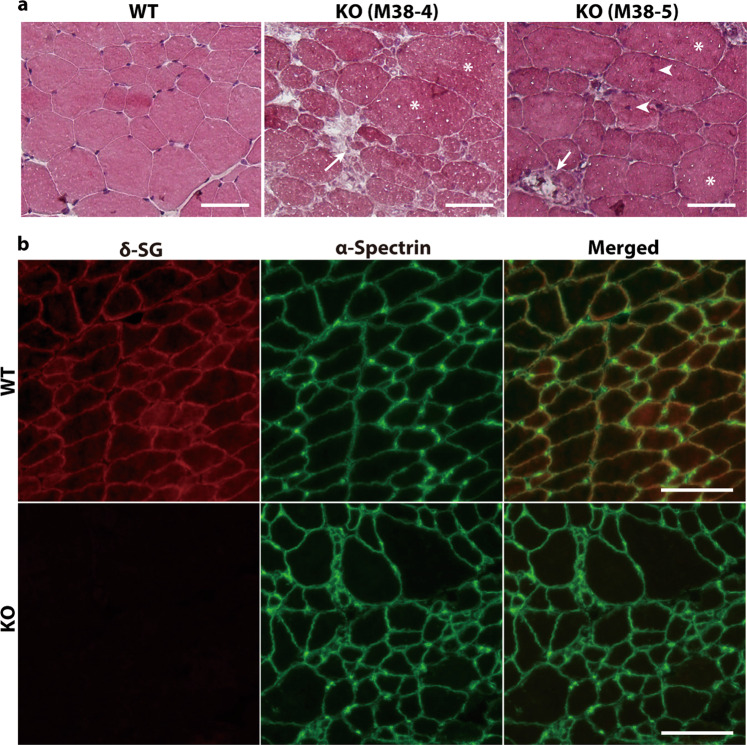
Histological analysis of the skeletal muscle in *SGCD*^−/−^ pigs. **a** HE-stained frozen sections of the skeletal muscles of WT and *SGCD*^−/−^ pigs (KO) at 8 weeks after birth. The skeletal muscles of week 8 *SGCD*^−/−^ animals showed necrosis (arrows), central nuclei (arrow heads), and hypertrophy (asterisks). Scale bars, 50 µm. **b** Skeletal muscle cryosections from WT and *SGCD*^−/−^ pigs (KO) at 8 weeks after birth were stained with antibodies against δ-SG (red) and the α-spectrin chain (green). Immunofluorescence analysis showed the lack of δ-SG expression in the muscle tissues of *SGCD*^−/−^ animals. Anti-spectrin antibodies were used to stain the sarcolemma. Scale bars, 50 µm.

Mutations in genes encoding SGs are responsible for the autosomal recessive forms of limb-girdle muscular dystrophies [32]. We observed serious degeneration of the skeletal muscles of each *SGCD*^−/−^ animal at 8 weeks after birth (M38-4 and M38-5; Fig. 4a). Muscular dystrophy is characterized by progressive muscular necrosis accompanied by regeneration, leading to variable muscle cell sizes. An increased myocyte number with central nucleation reflects regeneration. The degeneration process exceeded the regenerative potential such that replacement with connective and adipose tissues ensued [33]. These pathological features of muscular dystrophy-like lesions were observed in all mutant pigs but not in control animals (Fig. 4a).

Expression of the SG complex was analyzed using an immunofluorescence assay. In *SGCD*^−/−^ pigs, δ-SG was undetectable in the sarcolemma of the skeletal muscle fibers (Fig. 4b). The muscle cells of the *SGCD*^−/−^ clones exhibited irregular alignments and heterogeneous sizes (Fig. 4b).

**Fig. 5:**
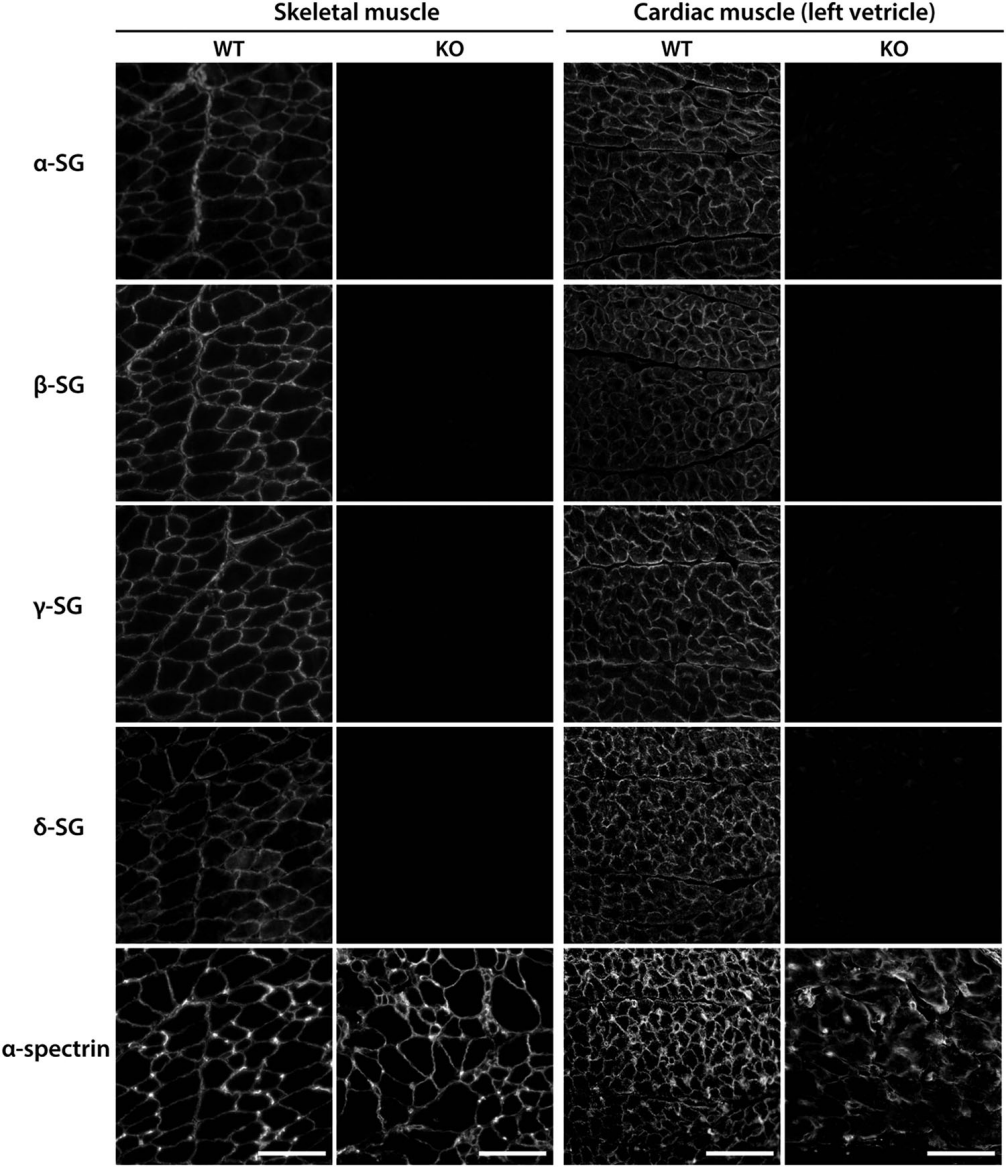
Immunofluorescence analysis of the SG complex in *SGCD*^−/−^ skeletal and cardiac muscle. Skeletal and cardiac muscle cryosections from WT and KO pigs at 8 weeks after birth were treated with antibodies against α-, β-, γ-, and δ-SG. The expression of SG isoforms was remarkably reduced in the *SGCD*^−/−^ animals. Scale bars, 50 µm.

**Fig. 6:**
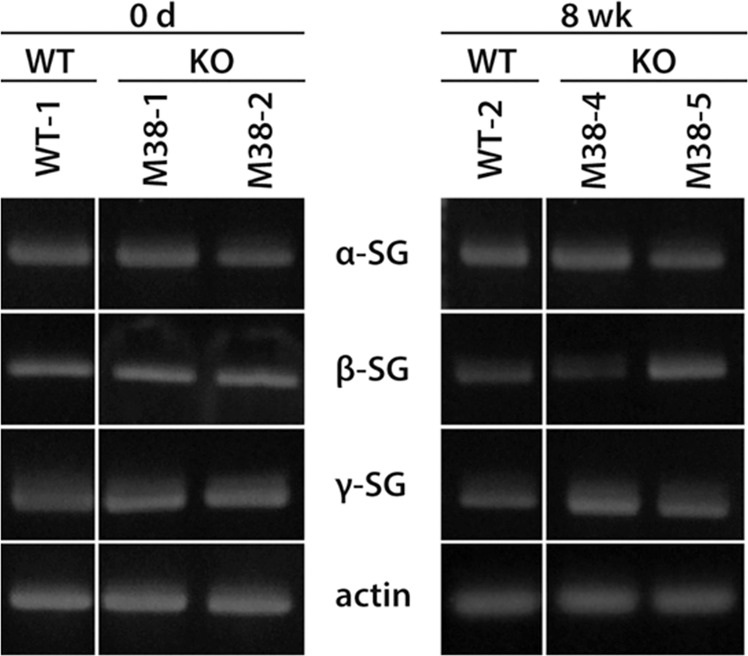
Analysis of the expression of SG mRNA in the skeletal muscle of *SGCD*^−/−^ pigs. Skeletal muscle microsomes from WT and *SGCD*^−/−^ pigs at day 0 or week 8 were analyzed using RT-PCR. Cropped gel images are shown. Original gels were run under the same experimental conditions.

The absence of one SG subunit affects the stability of the remaining SG subunits within the sarcolemma. For example, the remaining components of the SG complex are lost, or their levels are severely reduced, with the loss of one SG isoform [23, 34, 35]. Immunofluorescence analyses revealed that the levels of α-, β-, and γ-SG in the skeletal and cardiac sarcolemma of the *SGCD*^−/−^ pigs were remarkably lower than those of WT pigs (Fig. 5). We focused on α-spectrin, which lines the intracellular side of the plasma membrane, and found that its signal became blurred only in the cardiac muscle of KO clones (Fig. 5). These observations suggested that SG-deficient cardiac muscle cells were vulnerable to membrane damage when subjected to mechanical stress. Furthermore, when we analyzed SG expression in the LV, septum, and posterior wall, α-, β-, γ-, and δ-SG were undetectable in all parts of the myocardial tissues. In contrast, the levels of the respective mRNAs were comparable to those in WT pigs (Fig. 6). Taken together, these data indicated that posttranslational degradation might have led to the lack of detection of the SG isoforms.

### Functional and histological deterioration in the *SGCD*^−/−^ pig heart

**Fig. 7:**
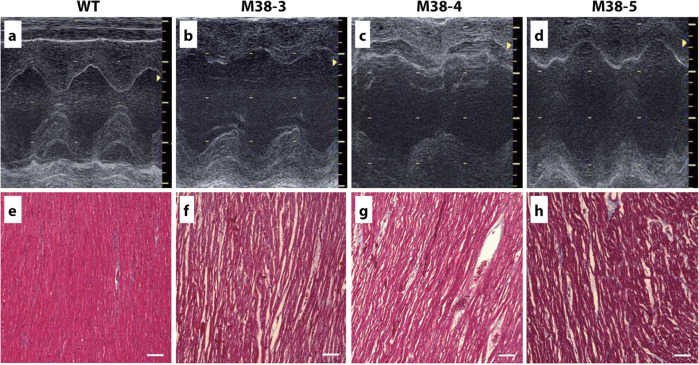
Cardiac function and histopathology of cloned *SGCD*^−/−^ pigs. Echocardiography was used to determine the dimensions and contractility of the LV of *SGCD*^−/−^ pigs (M-38-3, 4, 5) at 5 weeks of age compared with those of age-matched normal animals (WT). Compared with the WT pigs (**a**), all three *SGCD*^−^^/−^ animals showed dilated LV cavities during the systolic phase and thin LV walls, as shown in the M-mode echocardiographs (**b**–**d**). Masson’s trichrome staining of the cardiomyocytes suggested interstitial edema as indicated by the gaps between the cardiac muscle cells in the *SGCD*^−/−^ pigs (**f**–**h**) compared with those of WT animals (**e**). Scale bars in (**e**–**h**), 100 µm. **f** *SGCD*^−/−^ pig M38-3 at 7 weeks; **g**, **h** *SGCD*^−/−^ pigs M38-4 and M38-5 at 8 weeks.

The cardiac performance of the *SGCD*^−/−^ cloned pigs and age-matched WT animals was assessed using transthoracic echocardiography. The LVDs were greater in *SGCD*^*−/−*^ pigs than in WT pigs (*P* < 0.05; Table 1) while IVSth and PWth were lower in *SGCD*^−/−^ pigs than in WT pigs (*P* < 0.05). Moreover, the LV contractility was homogenously and globally decreased in the *SGCD*-null group, with a marked reduction of the ejection fraction (EF; *P* < 0.05; Fig. 7a–d).

**Table 1:** Cardiac performance of the *SGCD*^−/−^ cloned pigs.

	Age (weeks)	Body weight (kg)	LVDd (mm)	LVDs (mm)*	IVSth (mm)*	PWth (mm)*	LVEF (%)*
M38-3	5	5.6	27	21	4	4	49
M38-4	5	9.8	31	22	5	5	60
M38-5	5	9.3	28	18	3	4	67
WT 1	5	13.3	30	14	6	6	85
WT 2	5	14.5	27	13	7	6	85
WT 3	5	15.4	35	17	7	7	82

*M38-3, 4, 5* *SGCD*^−/−^, *WT* wild type, *LVDd* left ventricular diastolic dimension, *LVDs* left ventricular systolic dimension, *IVSth* interventricular septum thickness, *PWth* left ventricular posterior wall thickness, *LVEF* left ventricular ejection fraction.

*Values in the *SGCD*^−/−^ group significantly differ from those in the WT group (LVDs: *P* = 0.029, IVSth: *P* = 0.016, PWth: *P* = 0.013, LVEF: *P* = 0.036).

By performing histopathology on the cross-sections of cardiac tissues at the maximum short axis level after HE or Masson’s trichrome staining, HE-stained heart sections showed global hypertrophy of the LV wall with dilated left and right ventricular cavities in the *SGCD*^−/−^ animals, unlike that in WT animals. Moreover, Masson’s trichrome-stained heart sections (Fig. 7e–h) showed global and homogeneous gaps between cardiac muscle fibers throughout the LV walls in the KO clones, which suggested the presence of interstitial edema associated with advanced heart failure.

The serum levels of creatine kinase, troponin T, and atrial natriuretic peptide of the *SGCD*-KO pigs at 8 weeks (Table S4) were consistent with the morphological and histological findings for the cardiac and skeletal muscles, indicating that the development of these muscles was largely impaired.

### Generation of reproducible *SGCD*-mutant pig models

The above data are a proof-of-concept (POC) that biallelic frameshift mutation of the *SGCD* gene causes symptoms of genetic cardiomyopathy in pigs. Notably, the founder *SGCD*^−/−^ pigs were generated as somatic cell-cloned animals. Furthermore, the phenotype of the founder *SGCD*-mutant pigs might have been affected by epigenetic modification, which is prone to occur in somatic cell-cloned animals [36, 37]. Therefore, we investigated the phenotypes of *SGCD*^−/−^ pigs that had been generated via the reproduction process from a founder *SGCD*^−/+^ cloned pig.

Among the *SGCD*-mutant cell lines established for producing the *SGCD*^−/−^ cloned pigs, two lines (cell clones #34 with 2 bp deletion and #46 with 5 bp deletion) with monoallelic frameshift mutation were used to generate cloned pigs. Transfer of 95 and 91 SCNT embryos, respectively, derived from the nuclear donor cells #34 and #46 to two recipients, resulted in four and six male cloned pigs with the respective mutation type. These founder *SGCD*^−/+^ cloned pigs grew up normally beyond sexual maturity and mated with WT females to produce F1 *SGCD*^−^^/+^ offspring. Among the F1 offspring, *SGCD*^−/+^ males and females were mated with each other to generate F2 *SGCD*^−/−^ animals. Inbreeding was avoided by conducting mating between nonsibling males and females with different mutation types.

**Fig. 8:**
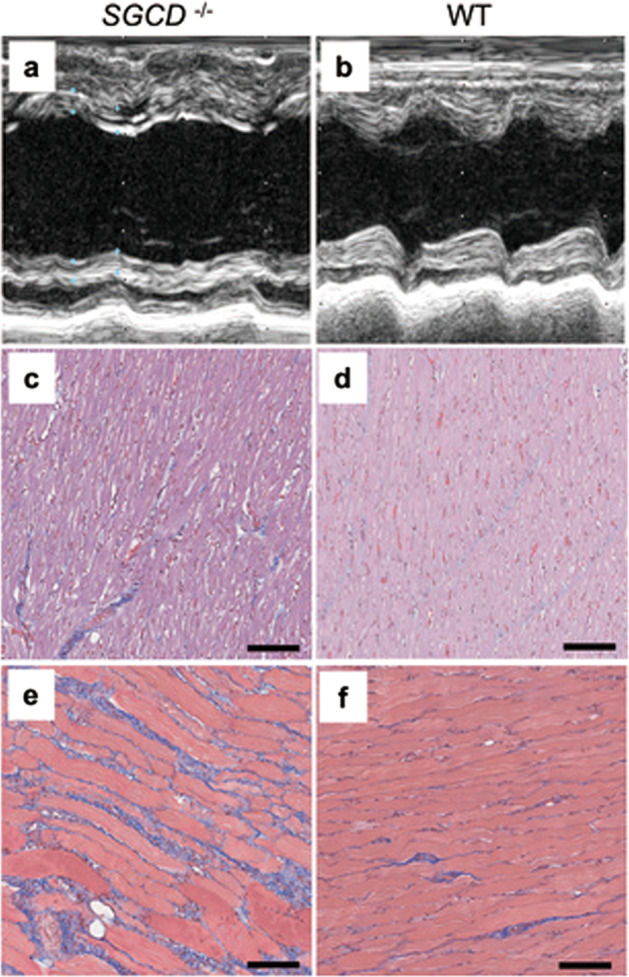
Cardiac and skeletal muscle pathology recapitulated in *SGCD*^−/−^ progeny. The *SGCD*^−/−^ progeny at 9 weeks (**a**, **c**, **d**) showed the pathological features of the cardiac and skeletal muscle observed in the cloned founder *SGCD*^−/−^ animals. These features included dilated LV cavities during the systolic phase detectable by echocardiography (**a**, **b**), interstitial edema as indicated by gaps between the cardiac muscle cells (**c**, **d**), and fibrotic regeneration in the skeletal muscle (**e**, **f**). **b**, **d**, **f** WT pig. HE (**c**, **d**) and Masson’s trichrome (**e**, **f**) staining. Scale bars, 100 µm.

The cardiac performance of the *SGCD*^−/−^ pigs was examined by conducting echocardiography on *SGCD*^−/+^ and *SGCD*^+/+^ littermates at 3, 6, and 9 weeks of age (Table 2). The LV ejection fraction of the *SGCD*^−/−^ pigs showed signs of deterioration at 6 weeks and significantly decreased at 9 weeks compared with that in the *SGCD*^−/+^ and *SGCD*^+/+^ siblings (55.0 ± 3.8 vs. 76.3 ± 2.9, 77.2 ± 2.9, respectively; *P* *<* 0.001). Such deterioration of cardiac performance was similar to that observed in the founder *SGCD*^−/−^ cloned pigs (Table 1). The LV tissue of the *SGCD*^−/−^ pigs exhibited homogenous gaps between muscle fibers (Fig. 8). The skeletal tissues of these animals showed pathological features, indicating serious muscular degeneration. All these pathological characteristics were similar to those observed in the *SGCD*^−/−^ cloned pigs.

**Table 2:** Cardiac performance of the progeny *SGCD*^−/−^ pigs.

Genotypes	Age (weeks)	LVDd (mm)	LV Ds (mm)	IVSth (mm)	PWth (mm)	LVEF (%)*
WT	3	13.4 ± 0.6	7.0 ± 0.6	2.4 ± 0.0	2.4 ± 0.2	85.2 ± 2.7
SGCD^+/−^	3	15.7 ± 1.6	8.1 ± 1.2	2.5 ± 0.4	2.7 ± 0.1	85.7 ± 1.1
SGCD^−/−^	3	14.8 ± 1.0	8.0 ± 0.8	2.6 ± 0.1	2.6 ± 0.2	83.7 ± 1.9
WT	6	24.3 ± 3.6	13.6 ± 3.4	4.3 ± 0.3	4.7 ± 0.4	82.7 ± 5.2
SGCD^+/−^	6	29.5 ± 1.5	17.1 ± 1.7	4.9 ± 0.5	5.1 ± 0.3	80.4 ± 2.7
SGCD^−^^/−^	6	28.2 ± 5.2	18.8 ± 4.6	4.4 ± 0.3	4.5 ± 0.2	70.4 ± 6.3
WT	9	30.6 ± 3.1	18.4 ± 1.3	5.1 ± 0.3	5.2 ± 0.9	77.2 ± 2.9
SGCD^+/−^	9	31.4 ± 0.8	19.3 ± 1.1	5.9 ± 1.1	6.4 ± 0.7	76.3 ± 2.9
SGCD^−/−^	9	27.5 ± 3.4	21.1 ± 3.2	4.3 ± 0.6	4.6 ± 0.5	55.0 ± 3.8

Two to 5 animals were used in each group.

*WT* wild-type, *SGCD*^+/−^ δ-SG gene heterozygous knockout pig, *SGCD*^−^^/−^ δ-SG gene homozygous knockout pig, *LVDd* left ventricular diastolic dimension, *LVDs* left ventricular systolic dimension, *IVSth* interventricular septum thickness, *PWth* left ventricular posterior wall thickness, *LVEF* left ventricular ejection fraction.

*Values in the *SGCD*^−^^/−^ group significantly differ from those in the WT and *SGCD*^+/−^ groups at 9 weeks (LVEF: *P* < 0.001).

## Discussion

The human gene encoding δ-SG comprises nine exons, as well as a 5′ untranslated region and three splice variants. In contrast, mouse *SGCD* comprises eight exons, and reports of splice variants are lacking. When we started this study in 2012, the exon−intron structure of porcine *SGCD* was not known. We revealed that pig *SGCD* includes eight exons, based on our sequence analysis and nucleotide sequence database search (GenBank/EMBL/DDBJ). Our result was verified later using the NCBI database (Gene ID, 100240724). Although further analysis is required to determine whether the transcript variants found in humans are encoded by this *SGCD*, the amino acid (a.a.) sequence (289 a.a.) of the predicted ORF of swine δ-SG identified according to the exon/intron boundaries is >90% similar to those in human (290 a.a., isoform 1) and mouse (289 a.a.) sequences, indicating that δ-SG is highly conserved among these species (pig vs. human: 96.90%; pig vs. mouse: 94.46%).

Spontaneous hamster models of δ-SG deficiency with the deletion of exon 2 [23, 35] are available for studying the genetic cardiomyopathy DCM. Exon 2, which harbors the start codon and transmembrane intracellular domains of SGCD, is targeted in a genetically modified mouse Duchenne muscular dystrophy (DMD) model [24, 25]. Therefore, we targeted exon 2 of pig *SGCD* and used a TALEN expression vector to create a homozygous gene KO. We combined genome editing of fibroblasts by using TALEN mRNA and SCNT to create *SGCD*-null pigs based on our previous successful use of the same techniques to efficiently generate interleukin-2 receptor (IL2R)-γ gene KO pigs [20].

Genome editing of porcine fibroblasts by using TALEN achieves efficiencies of monoallelic and biallelic mutations, ranging from 10 to 90% and 1 to 39%, respectively [38, 39]. The frequencies of monoallelic (36%) and biallelic (2%) editing achieved in the present study fell within the ranges described in previous studies [38, 39]. Recently, many researchers [40, 41], including us [42], have been successfully using clustered regularly interspaced short palindromic repeats and associated protein 9 (CRISPR/cas9) for generating genome-edited pigs. Nevertheless, herein, we showed that the use of TALEN mRNA and SCNT is a feasible option to produce gene KO in pigs.

The severe phenotypes of our *SGCD-*KO pigs provide genetic evidence that δ-SG, particularly in the SG complex, plays a pivotal role in maintaining muscle membrane integrity. Mutations in one SG gene are associated with the deficiency of the entire SG complex in the sarcolemma [43–45]. Herein, we showed that a complete deficiency of δ-SG accompanied a concomitant loss of the α-, β-, and γ-SG proteins, although normal levels of each transcript were maintained. These findings are consistent with those reported for spontaneous cardiomyopathy in hamsters of the BIO14.6 [23] and TO-2 [35] strains.

The cloned *SGCD*^−/−^ pigs exhibited progressive muscular dystrophy similar to that in human SG-deficient limb-girdle muscular dystrophy (LGMD) [46, 47]. Nigro et al. [32] reported that homozygous single-nucleotide deletions of *SGCD* cause LGMD, indicating that the correct assembly and proper transportation of the SG complex to the plasma membrane were hampered by δ-SG deficiency. Our results suggest that the mutation of *SGCD* dissociates the SG complex and thus contributes to progressive muscle degeneration.

Spectrin is known to be irreversibly cleaved by the calcium (Ca^2+^)-activated cytosolic cysteine protease calpain [48]. Previous studies have shown that defects in SG and the concomitant loss of the dystrophin-associated protein complex resulted in mechanical instability of the sarcolemma [49] and increased Ca^2+^ permeability, causing intracellular Ca^2+^ to chronically increase [50–53]. Bartoli et al. [53] also showed that basal calpain activity was elevated in SG-deficient SARCAFI mice. In addition, α-, β-, and γ-SG and dystrophin are preferentially hydrolyzed by calpain in vitro [54, 55]. These findings suggest that enhanced proteolysis, particularly by calpain, is a key factor in the progression of muscular dystrophy and/or cardiomyopathy.

In the present study, we showed that cloned *SGCD*^−/−^ swine exhibit systolic dysfunction similar to that found in human DCM. The DCM heart is consistently characterized by LV remodeling, manifesting as a dilated LV cavity, thinned LV wall, and reduced LV contraction [56]. Echocardiography showed a clear reduction in the LVEF of *SGCD*^−/−^ cloned pigs at 5 weeks of age compared with that of healthy animals. Deteriorated cardiac performance was also noted in the progeny *SGCD*^−/−^ pigs. However, the percentage decrease was slightly less than what is considered clinically impaired LV function in humans (LVEF 40%). This relatively low decrease may be explained by the young age of the animals (5 and 9 weeks); the symptoms of heart failure observed in patients with DCM might therefore also appear in aged *SGCD*^−/−^ pigs. Alternatively, the decrease in the reduction of LVEF in the *SGCD*^−/−^ clones may be attributed to physiological differences between pig and human hearts.

Genetic factors involved in DCM are found in 25–40% of patients [57–60]. Various mutations have been shown to occur in genes encoding cytoskeletal proteins involving dystrophin [61, 62] or its associated proteins, in addition to sarcomeric proteins [22]. For example, some familial and sporadic DCM cases have been attributed to mutations of the gene encoding δ-SG [24, 63], whereas sequence variants unrelated to these symptoms have also been reported [46].

Recent developments in next-generation therapeutics, such as stem cell therapy [64], cell sheet transplantation [65, 66], and gene therapy [35], offer opportunities for the treatment of advanced heart failure. Therefore, establishing large animal models that resemble human genetic cardiomyopathy is essential so that cardiac function can be thoroughly evaluated using the latest clinical imaging modalities [67, 68] in association with histological and biological evaluations. Such a model would be highly useful for exploring the feasibility, safety, and efficacy of therapeutic strategies, as well as for elucidating the underlying mechanisms of new treatments for genetic cardiomyopathy. In this study, the *SGCD*^−/−^ pigs prematurely died, confirming the vital role of δ-SG in maintaining normal cardiac function. Notably, O’Conner’s group identified human norepinephrine secretion as a highly heritable trait that is influenced by common genetic variations within the SGCD locus [69].

The severity of symptoms of the *SGCD*^−/−^ cloned pigs may indicate a synergistic effect of δ-SG deficiency and epigenetic changes that are specific to cloned animals. Various epigenetic alterations generated by the nuclear transfer of somatic cells are known to occur in cloned animals, including pigs [36, 37]. For example, dystrophin (*DMD*) KO pigs exhibit symptoms that are more severe than those of patients with DMD having the same genetic defect, and such pigs die prematurely [18]. Such differences between patients with DMD and *DMD*-KO pigs have been attributed to aberrant epigenetic alterations caused by somatic cell cloning [36]. Further investigation is necessary to identify epigenetic effects in cloned KO pigs. Nevertheless, generating somatic-cell-cloned pigs with homozygous gene KO is a straightforward way to gain a POC for the genetic mutation designed. In fact, in the present study, we generated only one litter of cloned pigs to verify the phenotype of the *SGCD*-KO. Alternatively, the injection of TALEN mRNA [28] or CRISPR/cas9 mRNA [29, 42] into pig embryos may be feasible for generating biallelic modifications if viable homozygous KO offspring can be selected after birth. However, this approach may involve the frequent production of animals with undesired mutations [42], which implies animal welfare concerns.

In conclusion, and to the best of our knowledge, the present study is the first to evidence that biallelic exon 2 frameshift mutation of *SGCD* in pigs leads to genetic cardiomyopathy. Furthermore, we showed that pigs heterozygously carrying the frameshift mutation were not affected by the disease, and the pathological conditions were reproduced in the progeny, which had inherited the mutations homozygously. These results present a feasible system for engineering a model pig for autosomal recessive cardiomyopathy, which includes acquisition of POC via cloned animals for the causal genetic mutation and proliferation of mutant animals as a research tool.

## Supplementary information

Supplementary Information
