# Correction: Pigs with δ-sarcoglycan deficiency exhibit traits of genetic cardiomyopathy

**DOI:** 10.1038/s41374-020-0414-7

**Published:** 2020-03-13

**Authors:** Hitomi Matsunari, Michiyo Honda, Masahito Watanabe, Satsuki Fukushima, Kouta Suzuki, Shigeru Miyagawa, Kazuaki Nakano, Kazuhiro Umeyama, Ayuko Uchikura, Kazutoshi Okamoto, Masaki Nagaya, Teruhiko Toyo-oka, Yoshiki Sawa, Hiroshi Nagashima

**Affiliations:** 1 Meiji University International Institute for Bio-Resource Research, Kawasaki, 214-8571 Japan; 2 Laboratory of Developmental Engineering, Department of Life Sciences, School of Agriculture, Meiji University, Kawasaki, 214-8571 Japan; 3 Department of Cardiovascular Surgery, Osaka University Graduate School of Medicine, Suita, 565-0871 Japan; 4 Department of Cardioangiology, Kitasato University, Sagamihara, 252-0375 Japan

**Keywords:** Genetic engineering, Genetic engineering

**Correction to: Laboratory Investigation**

10.1038/s41374-020-0406-7

This article was originally published under Nature Research’s License to Publish, but has now been made available under a [CC BY 4.0] license. The PDF and HTML versions of the article have been modified accordingly.

