# Supplementary material for: Pigs with δ-sarcoglycan deficiency exhibit traits of genetic cardiomyopathy
Source: Lab Invest. 2020 Feb 14;100(6):887–99. doi: 10.1038/s41374-020-0406-7 (PMC7280178; doi:10.1038/s41374-020-0406-7)
Supplement: Supplementary file 1 — Supplementary Information [file 41374_2020_406_MOESM1_ESM.pdf]

Supplementary Information

Pigs with δ-sarcoglycan deficiency exhibit traits of genetic cardiomyopathy

Hitomi Matsunari, Michiyo Honda, Masahito Watanabe, Satsuki Fukushima, Kouta Suzuki, Shigeru Miyagawa, Kazuaki Nakano, Kazuhiro Umeyama, Ayuko Uchikura, Kazutoshi Okamoto, Masaki Nagaya, Teruhiko Toyo-oka, Yoshiki Sawa, and Hiroshi Nagashima

a

```
ttccagcctctccacacacgcctcctggactgtgcggggttctcaccagccgctttgtgc   60

gaagaagacaagagagATGCCTCAGGAACAGTACACACACCACCGGAGCACCATGCCCAG   120
                 M  P  Q  E  Q  Y  T  H  H  R  S  T  M  P  S
CTCCGAGGGGCCACAGGTATACAAAGTAGGGATTTACGGCTGGCGGAAAAGATGCTTGTA   180
 S  E  G  P  Q  V  Y  K  V  G  I  Y  G  W  R  K  R  C  L  Y
TTTCTTTGTCCTACTCCTGATGATTTTAATACTGGTGAACTTGGCCATGACCATCTGGAT   240
 F  F  V  L  L  L  M  I  L  I  L  V  N  L  A  M  T  I  W  I
TCTCAAAGTCATGAACTTCACAATTGATGGAATGGGAAACTTGAGAATCACAGAAAAAGG   300
 L  K  V  M  N  F  T  I  D  G  M  G  N  L  R  I  T  E  K  G
TCTAAAGTTAGAAGGAGACTCAGAATTCCTACAACCTCTGTACGCCAAAGAAATCCAGTC   360
 L  K  L  E  G  D  S  E  F  L  Q  P  L  Y  A  K  E  I  Q  S
CCGACCAGGTAATGCCCTGTACTTCAAATCTGCCAGAAATGTTACTGTGAACATCCTCAA   420
 R  P  G  N  A  L  Y  F  K  S  A  R  N  V  T  V  N  I  L  N
TGAACAGACTAAAGTGCTAACTCAGCTGATAACAGGTCCAAATGCCATAGAAGCTTATGG   480
 E  Q  T  K  V  L  T  Q  L  I  T  G  P  N  A  I  E  A  Y  G
CAAAAAGTTTGAAGTAAAGACGGTTTCTGGAAAATTGCTCTTCTCTGCAGATAACAATGA   540
 K  K  F  E  V  K  T  V  S  G  K  L  L  F  S  A  D  N  N  E
AGTCGTAGTGGGAGCTGAAAGATTGAGAGTTTTAGGAGCAGAGGGCACAGTGTTCCCTAA   600
 V  V  V  G  A  E  R  L  R  V  L  G  A  E  G  T  V  F  P  K
ATCTATAGAGACACCTAATGTCAGGGCAGACCCCTTCAAGGAACTAAGGTTGGAATCCCC   660
 S  I  E  T  P  N  V  R  A  D  P  F  K  E  L  R  L  E  S  P
CACCCGGTCTCTGGTGATGGAAGCCCCAAAAGGAGTAGAAATAAATGCAGAAGCCGGCAA   720
 T  R  S  L  V  M  E  A  P  K  G  V  E  I  N  A  E  A  G  N
TATGGAAGCCACCTGCAGAACAGAGCTGAGACTGGAGTCCAAAGATGGAGAGATTAAGTT   780
 M  E  A  T  C  R  T  E  L  R  L  E  S  K  D  G  E  I  K  L
AGATGCTGCGAAAATCAAACTACCCAGATTGCCTCACGGATCCTACACACCCGCGGGAAC   840
 D  A  A  K  I  K  L  P  R  L  P  H  G  S  Y  T  P  A  G  T
GAGGCAGAAGGTCTTCGAGATCTGCGTTTGTGCCAACGGGAGATTATTCCTGTCACAGGC   900
 R  Q  K  V  F  E  I  C  V  C  A  N  G  R  L  F  L  S  Q  A
AGGAACTGGTTCCACTTGTCAGATAAACACAAGTGTCTGCCTGTGAgagactatccatag   960
 G  T  G  S  T  C  Q  I  N  T  S  V  C  L  *
tggacactgtcgcagcataaaggcctttttggctttagaccctggctgccagctatttt   1020

tactataacacagaaagcctatcaaagaccttttgtgtgtgtgtgtgc             1068
```

b

Transmembrane domain (Predicted)

```
        ****** ******* * **  ****************************************
Human  MMPQEQYTHHRSTMPGSVGPQVYKVGIYGWRKRCLYFFVLLLMILILVNLAMTIWILKVM
Mouse   MPQEQYSHHRSTMPSSEGPHIYKVGIYGWRKRCLYFFVLLLMILILVNLAMTIWILKVM
  Pig   MPQEQYTHHRSTMPSSEGPQVYKVGIYGWRKRCLYFFVLLLMILILVNLAMTIWILKVM

       ************************************ ********************** ***
Human  NFTIDGMGNLRITEKGLKLEGDSEFLQPLYAKEIQSRPGNALYFKSARNVTVNILNDQTK
Mouse  NFTIDGMGNLRITEKGLKLEGDSEFLQPLYAKEIKSRPGNALYFKSARNVTVNILNDQTK
  Pig  NFTIDGMGNLRITEKGLKLEGDSEFLQPLYAKEIQSRPGNALYFKSARNVTVNILNEQTK

       ***** *** * ***** ***************  **************************
Human  VLTQLITGPKAVEAYGKKFEVKTVSGKLLFSADNNEVVVGAERLRVLGAEGTVFPKSIET
Mouse  VLTQLVTGPKAVEAYGKRFEVKTVSGKLLFSADDSEVVVGAERLRVLGAEGTVFPKSIET
  Pig  VLTQLITGPNAIEAYGKKFEVKTVSGKLLFSADNNEVVVGAERLRVLGAEGTVFPKSIET

       ****************************************** ** *****************
Human  PNVRADPFKELRLESPTRSLVMEAPKGVEINAEAGNMEATCRTELRLESKDGEIKLDAAK
Mouse  PNVRADPFKELRLESPTRSLVMEAPKGVEINAEAGNMEAICRSELRLESKDGEIKLDAAK
  Pig  PNVRADPFKELRLESPTRSLVMEAPKGVEINAEAGNMEATCRTELRLESKDGEIKLDAAK

       * ***** ***** ******** *************** *************
Human  IRLPRLPHGSYTPTGTRQKVFEICVCANGRLFLSQAGAGSTCQINTSVCL
Mouse  IKLPRLPRGSYTPTGTRQKVFEVCVCANGRLFLSQAGTGSTCQINTSVCL
  Pig  IKLPRLPHGSYTPAGTRQKVFEICVCANGRLFLSQAGTGSTCQINTSVCL
```

Supplementary Fig. S1. cDNA and amino acid sequences for porcine *SGCD*.
(a) Pig *SGCD* cDNA and the encoded amino acid sequence. The sequence is available from GenBank under accession number NM_001144123. The exon/intron boundaries (exons 2–6) are indicated by the inverted black triangle. *Asterisks* indicate termination codons. Uppercase and lowercase letters represent coding and untranslated regions, respectively. **(b)** Amino acid sequences of pig, human, and mouse SGCD (GenBank accession numbers: NP_000328, NP_036021, and NP_001137595, respectively). The predicted transmembrane domain is indicated by the shadowed box[1]. *Asterisks* indicate identical amino acids.

Supplementary Table S1. The primer sets and PCR conditions used in the genome walking.

Primers	Sequence (5' – 3')	Position	
SGCD01	gatttacggctggcggaaaagatgcttg	Exon2	sense primer
SGCD02	gggactggatttctttggcgtacagagg	Exon3	antisense primer
SGCD03	tgggaaacttgagaatcacagaaaaagg	Exon3	sense primer
SGCD04	tcagctgagttagcactttagtctgttc	Exon4	antisense primer
SGCD05	gccctgtacttcaaatctgccagaaatg	Exon4	sense primer
SGCD06	aactctcaatctttcagctcccactacg	Exon5	antisense primer
SGCD07	tatggcaaaaagtttgaagtaaagacgg	Exon5	sense primer
SGCD08	ccacttaatcaatgcaaaccatagtcag	Exon6	antisense primer
SGCD09	actggtgaacttggccatgaccatctgg	Exon2	sense primer
SGCD10	attctgagtctccttctaactttagacc	Exon3	antisense primer
SGCD11	ctaaagttagaaggagactcagaattcc	Exon3	sense primer
SGCD12	ttgaggatgttcacagtaacatttctgg	Exon4	antisense primer
SGCD13	ttactgtgaacatcctcaatgaacagac	Exon4	sense primer
SGCD14	gacttcattgttatctgcagagaagagc	Exon5	antisense primer
SGCD15	aaattgctcttctctgcagataacaatg	Exon5	sense primer
SGCD16	ccttgaaggggtctgccctgacattagg	Exon6	antisense primer
AP1	GTAATACGACTCACTATAGGGC		
AP2	ACTATAGGGCACGCGTGGT		

The first PCR amplification using PrimeSTAR HS DNA polymerase (Takara Bio) was performed under the following conditions: denaturation at 98 °C for 60 s, 7 cycles of amplification at 98 °C for 25 s and 68 °C for 3 min, 32 cycles of amplification at 98 °C for 25 s and 64 °C for 3 min, and a final elongation step at 64 °C for 7 min. The nested PCR amplification was performed using PrimeSTAR HS DNA polymerase using the following conditions: denaturation at 98 °C for 60 s, followed by 5 cycles of amplification at 98 °C for 25 s and 68 °C for 3 min, 20 cycles of amplification at 98 °C for 25 s and 64 °C for 3 min, and a final elongation step at 64 °C for 7 min. Amplification conditions: Preheating at 95 °C for 1 min, followed by 30-40 cycles at 94 °C for 20 s, 58 °C (for *α-, β-,* and *γ-SG*) or 55 °C (for *SGCD* and *ACTB*) for 30 s, and 72 °C for 90 s.

Supplementary Table S2. Primer sequences for genes confirmed by RT-PCR.

	Forward primer	Reverse primer
α-SG	TCCCATACCAGGCTGAGTTC	CATAGCAGGACAGCAGTGGA
β-SG	CATCACTGGCAACAATCAGC	CACAGGGGTTGTCTGAGGTT
γ-SG	GCGCTGCCTCTACTTATTCG	AAGTTTCGATGTGCGTAGGG
δ-SG	GCTTTGTGCGAAGAAGACA	AAAGGCCTTTATGCTGCGAC
Actin	TCTGGCACCACACCTTCTACA	GCCACGTAGCACAGCTTCTC

Supplementary Table S3. *SGCD* mutations induced by TALEN mRNA in PFF cells.

Target	Cell clones analyzed	Event	Mutation type	No. of cell clones (%)	Cell clones with frameshift mutations (%)
SGCD	177	Biallelic	Deletion	4 (2.3)	2 (1.1)
			Insertion	0 (0)	0 (0)
			Complex*	0 (0)	0 (0)
		Monoallelic	Deletion	36 (20.3)	22 (12.4)
			Insertion	5 (2.8)	5 (2.8)
			Complex*	4 (2.3)	2 (1.1)
			ND**	18 (10.2)	-
			Total	67 (37.9)	31 (17.5)

* deletions, insertions, and/or substitutions
**not determined in detail

Supplementary Table S4. Serum biochemical profile of the *SGCD*-KO cloned pigs.

		SGCD-KO pigs		Control WT pig
		M38-4	M38-5	(n = 1)
CK (U/L)		85,500	72,350	1,199
CK isozyme (%)	BB	1	1	9
	MB	3	2	2
	MM	96	97	89
TnT (ng/mL)		0.193	0.300	0.010
ANP (pg/mL)		73.6	215.0	24.0

Serum level of creatine kinase (CK), troponin T (TnT), and atrial natriuretic peptide (ANP) of the *SGCD*-KO pigs at 8 weeks of age was quantitatively analyzed. Creatine kinase was markedly greater in the *SGCD*-KO pigs than in the WT pigs, whereas the proportions of skeletal muscle isozyme in the serum creatine kinase were more than 85% in both *SGCD*-KO and WT pigs. In addition, the serum levels of troponin T and atrial natriuretic peptide were substantially greater in the *SGCD*-KO pigs than the WT pig.

Reference

1 Jung D, Duclos F, Apostol B, Straub V, Lee JC, Allamand V, et al. Characterization of δ-sarcoglycan, a novel component of the oligomeric sarcoglycan complex involved in limb-girdle muscular dystrophy. J Biol Chem 1996;271:32321–32329.
